# Profound deficits in hippocampal synaptic plasticity after traumatic brain injury and seizure is ameliorated by prophylactic levetiracetam

**DOI:** 10.18632/oncotarget.23923

**Published:** 2018-01-04

**Authors:** Yuan-Hao Chen, Tung-Tai Kuo, Eagle Yi-Kung Huang, Barry J. Hoffer, Yu-Ching Chou, Yung-Hsiao Chiang, Hsin-I Ma, Jonathan P. Miller

**Affiliations:** ^1^ Department of Neurological Surgery, Tri-Service General Hospital, National Defense Medical Center, Taipei, Taiwan, R.O.C; ^2^ Graduate Institute of Computer and Communication Engineering, National Taipei University of Technology, Taipei, Taiwan, R.O.C; ^3^ Department of Pharmacology, National Defense Medical Center, Taipei, Taiwan, R.O.C; ^4^ Department of Neurosurgery, Case Western Reserve University School of Medicine, Cleveland, Ohio, USA; ^5^ School of Public Health, National Defense Medical Center, Taipei, Taiwan, R.O.C; ^6^ Division of Neurosurgery, The Ph.D. Program for Neural Regenerative Medicine, Taipei Medical University Hospital, Taipei, Taiwan, R.O.C

**Keywords:** short-term presynaptic plasticity, traumatic brain injury, seizures, hippocampus, long-term potentiation

## Abstract

**Aim:**

To determine the precise effects of post-traumatic seizure activity on hippocampal processes, we induced seizures at various intervals after traumatic brain injury (TBI) and analyzed plasticity at CA1 Schaffer collateral synapses.

**Material and Methods:**

Rats were initially separated into two groups; one exposed solely to fluid percussion injury (FPI) at 2 Psi and the other only receiving kainic acid (KA)-induced seizures without FPI. Electrophysiological (ePhys) studies including paired-pulse stimulation for short-term presynaptic plasticity and long-term potentiation (LTP) of CA1 Schaffer collateral synapses of the hippocampus for post-synaptic function survey were followed at post-event 1 hour, 3 and 7 days respectively. Additional rats were exposed to three seizures at weekly intervals starting 1 week or 2 weeks after TBI and compared with seizures without TBI, TBI without seizures, and uninjured animals. An additional group placed under the same control variables were treated with levetiracetam prior to seizure induction. The ePhys studies related to post-TBI induced seizures were also followed in these additional groups.

**Results:**

Seizures affected the short- and long-term synaptic plasticity of the hippocampal CA3-CA1 pathway. FPI itself suppressed LTP and field excitatory post synaptic potentials (fEPSP) in the CA1 Schaffer collateral synapses; KA-induced seizures that followed FPI further suppressed synaptic plasticity. The impairments in both short-term presynaptic and long-term plasticity were worse in the rats in which early post-TBI seizures were induced than those in which later post-TBI seizures were induced. Finally, prophylactic infusion of levetiracetam for one week after FPI reduced the synaptic plasticity deficits in early post-TBI seizure animals.

**Conclusion:**

Our data indicates that synaptic plasticity (i.e., both presynaptic and postsynaptic) suppression occurs in TBI followed by a seizure and that the interval between the TBI and seizure is an important factor in the severity of the resulting deficits. Furthermore, the infusion of prophylactic levetiracetam could partially reverse the suppression of synaptic plasticity.

## INTRODUCTION

Traumatic brain injury (TBI) frequently leads to severe and persistent neurological deficits that can result in considerable morbidity. In addition to neurological deficits, TBI often leads to headache, psychological impairment, and cognitive impairment [1–3], and the combined effects of these disorders produce a substantial burden on society. Temporary or permanent memory loss is seen in many head injury patients [4] and can lead to long-term deficits in learning and cognition [5–7]. However, the nature and extent of damage is variable, and long-term recovery can be difficult to predict. There is presently no known method of preventing memory deficits after TBI or treating them once they occur.

Seizures often occur after TBI, and TBI is a major risk factor for subsequent development of epilepsy [8–10]. There is a strong correlation between posttraumatic epilepsy (PTE) and the loss of brain volume after head injury [11]. Since seizures and TBI are independently associated with memory and learning deficits, it is possible that the two effects may be synergistic such that seizures exacerbate TBI-related physiological dysfunction. There is also evidence that the interval between a TBI and a post-TBI seizure may impact both the severity of TBI comorbidities and epileptogenesis [12, 13], and early seizures (defined as occurring within one week after injury) [14] may be associated with worse memory outcome than seizures that occur remotely. If this is the case, aggressive seizure prophylaxis may be important for improved outcome.

If the combined effect of TBI and seizures on hippocampal processes is greater than either alone, a better understanding of the underlying mechanisms might provide guidance for further clinical therapeutic research efforts. In this study, we report the changes in hippocampal electrophysiological correlates of plasticity of seizures at various intervals after TBI, as well as the capacity of prophylactic antiepileptic medications to ameliorate these effects.

The animals used in this study are a subset of animals used in our previous publication [13]. A diagrammatic representation of manipulations, and treatment times of the various animal groups for this study are shown in Supplementary Data 1.

## RESULTS

### Short-term plasticity of hippocampal CA1-CA3 Schaffer collateral synapses is affected by KA-induced seizures and by fluid percussion in injury (Figure 1)

**Figure 1:**
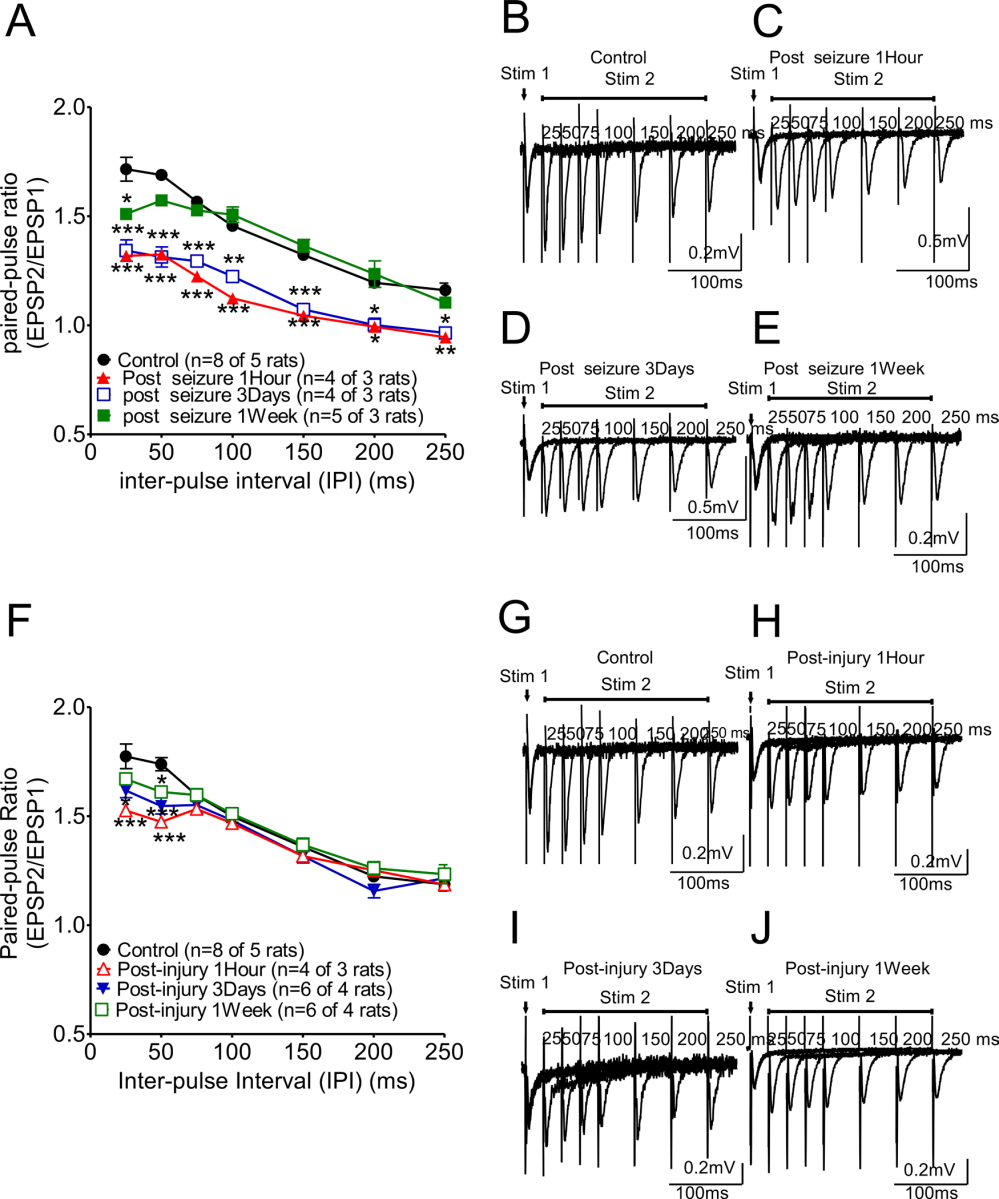
Paired-pulse ratio changes in hippocampal CA1 Schaffer collateral synapses following KA-induced seizures or fluid percussion injury **(A)** The ratio was markedly suppressed initially (one hour after KA-induced seizures; red triangle curves) and this effect persisted at three days after KA-induced seizures (open blue box curve) compared with the ratios of control animals (black circle curve). This suppression of this ratio was partially reversed at one week after KA-induced seizures (green box curve). A representative trace from each group is shown in panels **(B)** to **(E)**. **(F)** The paired-pulse facilitation after high frequency stimulation (IPI ≤ 75ms) was suppressed at 2Psi in FPI animals while PPR was not affected with longer IPI. This PPR suppression was significant at post-injury 1 hour, and reversed gradually at post-injury 3 days and one week (Figure 1G-1J).

**Figure 2:**
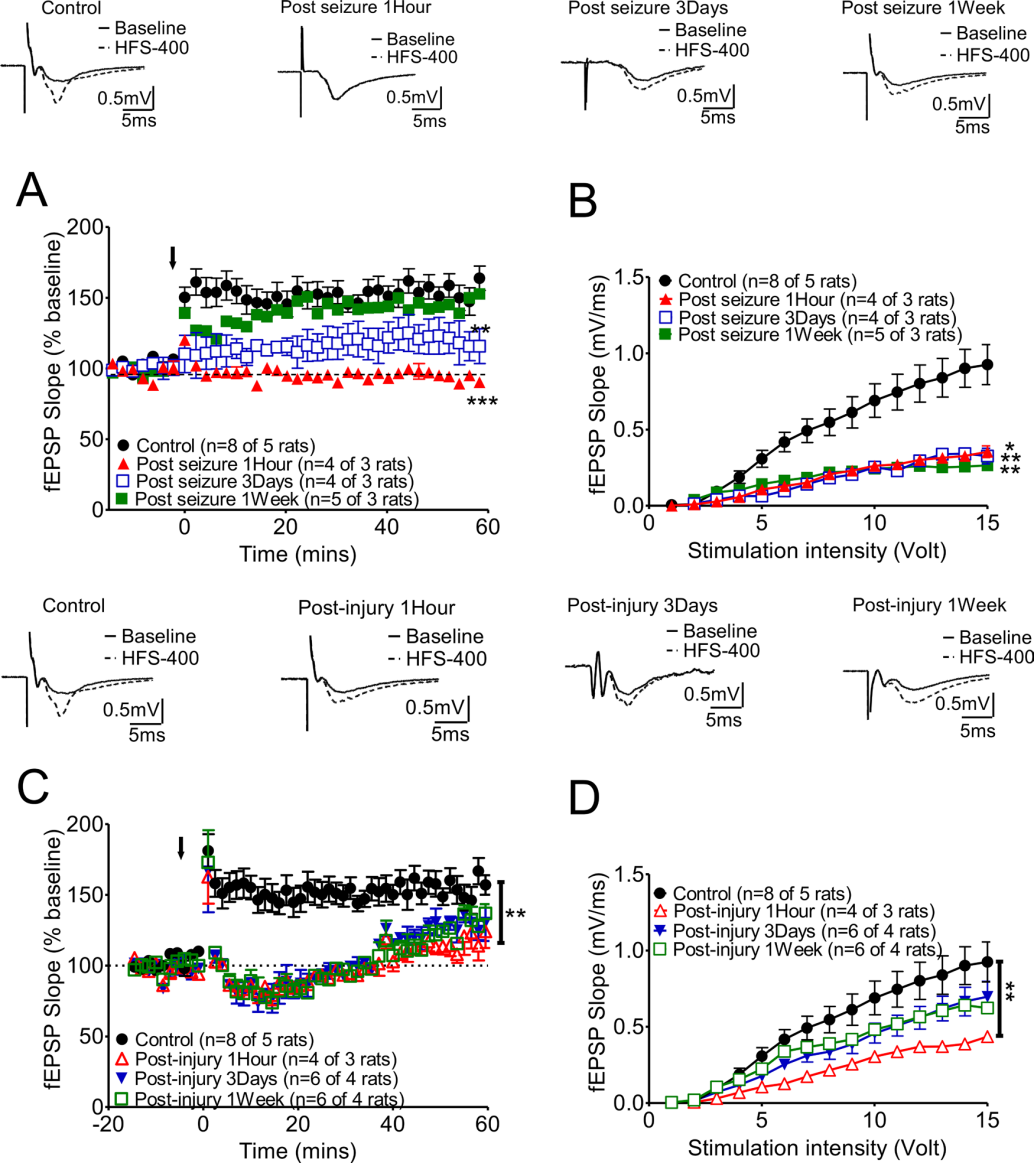
Long-term synaptic plasticity of hippocampal CA3-CA1 Schaffer collateral synapses was affected either by KA-induced seizures or fluid percussion injury alone, and these changes varied at different recording times **(A)** Marked suppression of LTP was found at 3 days after KA-induced seizures, while this suppression was partially reversed at 7 days after seizures. A representative trace of each group is shown in the upper panels. **(B)** The slopes of fEPSPs were generally suppressed after KA-induced seizures. **(C)** After 2 Psi FPI, the plasticity was impaired in post-1 hour and 3 days animals and mild potentiation was found at the end of the recording time (Post-FPI 1 hour: open red triangles; Post-FPI 3 days: solid blue triangles). LTP could be induced one week after 2 Psi FPI (Open green squares). **(D)** The slope of fEPSPs were also suppressed by 2Psi FPI initially (1 hour after FPI) and then recovered gradually.

To check the effects of seizures on short-term plasticity only, we measured the paired-pulse ratio (PPR) evoked by different stimulation inter pulse intervals (IPI) (from 10msec to 250msec) in hippocampal CA1 Schaffer collateral synapses after KA-induced seizures with and without FPI. The PPR was affected at very early stages after KA-induced seizures (Figure 1A-1E, one hour after KA-induced seizure; red triangle curve) and was suppressed at three days after KA-induced seizures (blue open box curve) compared with the PPR in the control animals (black circle curve). PPR recovered at one week after KA-induced seizures (green black box curve). Animals given FPI without KA also showed suppression of PPR with gradual recovery by 1 week (Figure 1G-1J). Of note, the combination of TBI and KA seizures showed a significantly greater deficit and longer recovery time (see Figure 2A).

### Hippocampal CA1-CA3 Schaffer collateral LTP formation was also impaired by KA-induced seizures initially and then recovered one week later (Figure 3)

To determine the impact of seizures on long-term synaptic plasticity, we analyzed the LTP at hippocampal CA3-CA1 Schaffer collateral synapses at one hour, 3 days, and one week after KA-induced seizures. LTP was affected by KA-induced seizures, and the degree of change varied at different recording times. Initially, LTP was totally suppressed at one hour after seizures (Figure 2A, red triangle). Marked suppression of LTP was found at 3 days after KA-induced seizures (blue open box curve), while this suppression partially recovered at 7 days after seizures (green black box curve). The representative traces from each group are shown in the upper panels of Figure 2. The slopes of the fEPSPs in the KA-induced seizure groups were generally suppressed compared to those of the control group (Figure 2B). FPI also altered LTP with gradual recovery (Figure 2C, 2D). Of note, the combination of TBI and KA seizures showed a much longer recovery time and greater deficit (see Figure 4A, 4D).

**Figure 3:**
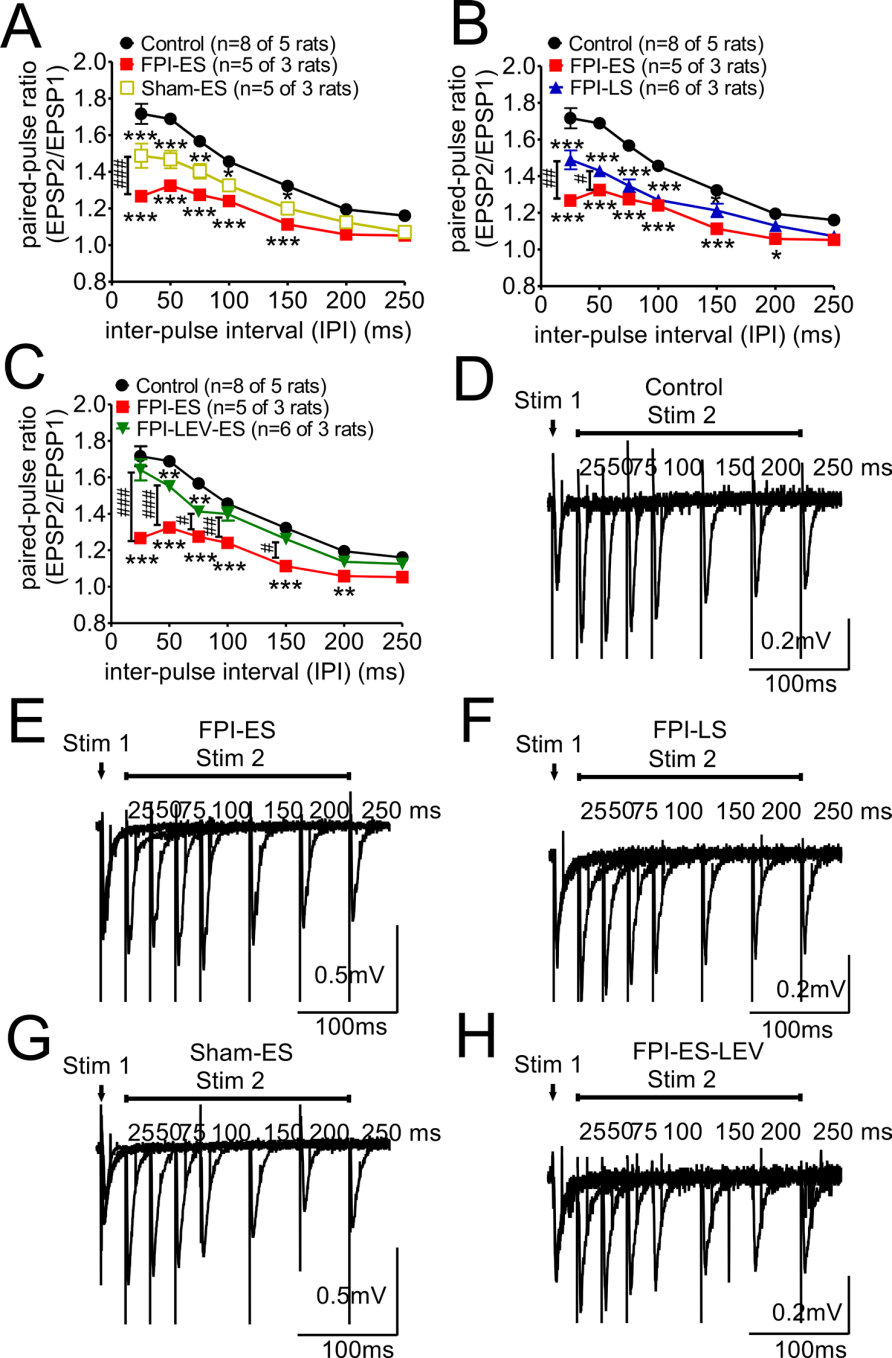
Paired-pulse stimulation responses, impaired in the post-FPI seizure animals, could be partially reversed by prophylactic administration of LEV Under our experimental conditions (Mg ^2+^: 1.5 mM and Ca ^2+^: 2.4 mM and the initial evoked potentials being 30% of the maximal response), the responses were higher in second evoked potentials with a short interval, which is referred as PPF (paired-pulse facilitation). **(A)** Regardless of whether the animals were injured or not, the extent of PPF in the seizure animals was less than that of the control animals. In addition, profound suppression of PPF was found in the injured animals subjected to seizures (solid red squares). **(B)** PPF was suppressed in both early and late post-injury seizure animals compared with control animals. The suppression of PPF in the early seizure group was more marked (solid red squares) than that in the later seizure group of injured animals (solid blue triangles) **(C)** The suppression of PPF in the early seizure group of injured animals (FPI-ES, solid red squares) could be reversed by the prophylactic injection of LEV (FPI-LEV-ES, open green triangles). **(D-H)** The representative wave forms for paired-pulse stimulation of each group are shown.

**Figure 4:**
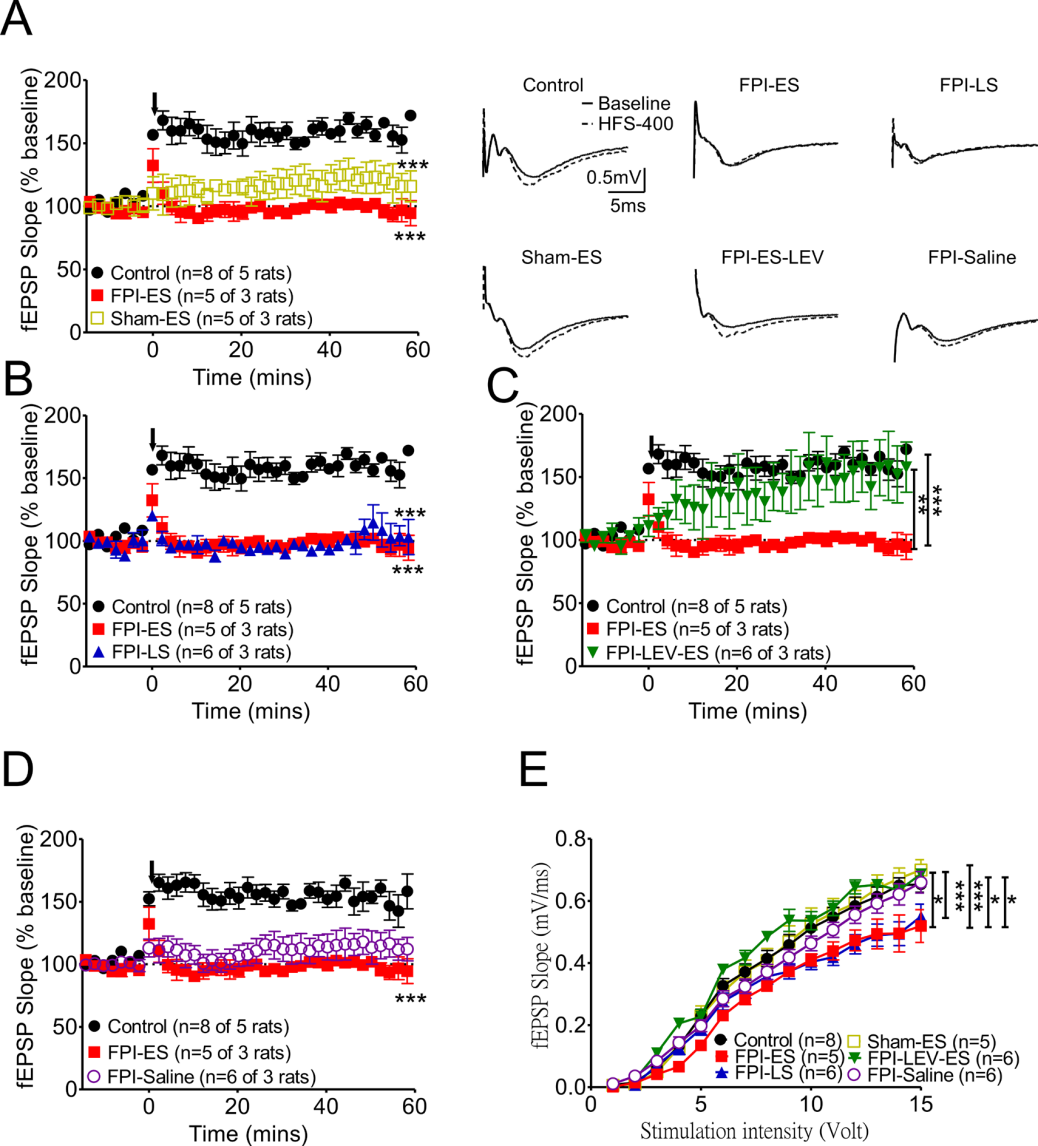
After brain injury, the suppression of synaptic plasticity as manifested by LTP in the hippocampus in seizure animals was prominent **(A)** Synaptic plasticity at CA3-CA1 synapses was compared in control, FPI-ES, and sham–SE groups. LTP, readily induced in control animals, was virtually absent in seizure animals with brain injury (FPI-ES group, red squares). **(B)** After brain injury, neither the early seizure (FPI-ES) nor late seizure (FPI-LS, blue triangles) groups manifested LTP in fEPSPs from the CA3-CA1 pathway. **(C)** If the brain injury animals received prophylactic levetiracetam infusion for one week after injury, synaptic plasticity was partially restored (FPI-LEV-ES group, inverted green triangles). **(D)** LTP formation in the FPI-only group (FPI-saline group, open purple circles) was impaired, being significantly suppressed by early seizure induction after FPI (FPI-ES group, red squares). **(E)** The evoked potentials used in the LTP experiments were 45~50% of the maximum response to stimulation after we performed input-output (I/O) curves at each stimulation site in each hippocampal brain slice. ^*^:P<0.05, ^***^:P<0.001 Control vs other groups, ^##^:P<0.01, ^###^:P<0.001 Sham+ES vs other groups, $:P<0.05 FPI+Saline vs FPI+ES.

### Paired-pulse stimulation responses were impaired in post-FPI + KA seizure animals but could be partially reversed by prophylactic injection of LEV (Figure 3)

We next assessed the effect of FPI combined with seizures on short-term plasticity. By applying paired-pulse stimulation at different stimulation intervals (from 10 to 250 msec) to each brain slice, the paired-pulse ratio (PPR) was calculated. Under our experimental conditions (Mg ^2+^: 1.5 mM and Ca ^2+^: 2.4 mM and the initial evoked potentials being 30% of the maximal response), the responses were higher for second evoked potentials with a short interval, which is referred as PPF (paired-pulse facilitation). Regardless of whether the animal was injured or not, the extent of PPF in the seizure animals was less than that in the control animals (Figure 3A), whereas a more profound suppression of PPF was found in the injured animals subjected to seizures (Figure 3A, red solid squares). Although PPF was suppressed in both post-injury early and late onset seizure animals in comparison to the control animals, the suppression was greater in the early seizure group (Figure 3B, red solid squares) than in the late seizure group of injured animals (Figure 3B, blue solid triangles). When prophylactic LEV was administered after injury, the suppression of PPF in the early seizure group of injured animals (Figure 3C, FPI-ES; red solid squares) could be reversed (Figure 3C, FPI-LEV-ES, green open triangles). The representative wave forms for paired-pulse stimulation for each group are shown in Figure 3D-3H.

### Post-TBI early seizures profoundly impaired LTP formation compared with late post-TBI seizures (Figure 4)

Next, to determine the effect of brain injury and seizure on long-term synaptic plasticity, the long-term potentiation of CA1 Schaffer collateral synapses was assessed in the FPI groups with early or late onset of seizures induced by KA. Our data indicate that the suppression of synaptic plasticity, manifested by LTP in the hippocampus in seizure animals, was prominent (Figure 4A). LTP, which was readily induced in control animals, was virtually absent in seizure animals after brain injury (Figure 4A FPI-ES group, red square). After brain injury, LTP could not be induced in either the early seizure (Figure 4B, FPI-ES) or late seizure (Figure 4B, FPI-LS, blue triangle) groups. However, when prophylactic levetiracetam was administered by pump infusion for one week after injury, synaptic plasticity was partially restored (Figure 4C, FPI-LEV-ES group, reverse green triangles). LTP formation in the FPI-only group (Figure 4D, FPI-saline group, open purple circle) was impaired, but these impairments were more profound with early seizure induction after FPI (Figure 4D, FPI-ES group, red squares). The evoked potentials used in the LTP experiments were 45~50% of the maximum response to stimulation after we generated input-output (I/O) curves for each stimulation site in the hippocampal brain slices. The I/O curve of the FPI-only group (FPI-saline group, open purple circles) was shifted to the right (i.e. moderately suppressed by the injury), and the I/O curves of the injured animals with early onset seizure (Figure 4E, FPI-ES, red squares) or late onset seizure (Figure 4E, FPI-LS, blue triangles) were suppressed more significantly and shifted further to the right compared with the seizure-only (Figure 4E, sham-ES, open yellow squares) and control groups (Figure 4E, black circles).

## DISCUSSION

TBI has been implicated in memory loss and other deficits [5, 15, 16], and seizures are also known to contribute to learning impairments [17, 18]. Memory and learning may be impaired by both TBIs and seizures; therefore, in patients with posttraumatic seizures, deficits to memory and learning processes may be profound. The current study sought to test this hypothesis by using FPI and KA-induced seizure animal models to determine the mechanisms underlying such changes in order to better inform future clinical applications.

In this study, we measured presynaptic as well as postsynaptic transmission in the hippocampus through the use of short-term and long-term synaptic plasticity experiments conducted with rats subjected to FPI followed by early or late KA-induced seizures.

First, in the short-term plasticity experiment, PPRs were markedly suppressed at the acute stage (at 1 hour and 3 days after KA-induced seizure), and this suppression partially recovered (Figure 1A). In the long-term plasticity experiment, the seizures showed acute effects on LTP. The suppression of LTP could be observed at 3 days after a seizure, but this suppression partially recovered at one week after the seizure (Figure 2). However, the input-output curves (Figure 2B, I/O curve), representing the slope of fEPSPs with different stimulation intensities from 1 to 15 volts, in seizure animals were all shifted significantly to right even at one week after seizures. This shift to the right may relate to impaired glutamaturgic transmission. Biochemical findings have demonstrated that injury leads to significantly smaller N-methyl-D-aspartate (NMDA) potentials and glutamate-induced excitatory currents, increased dendritic spine size, and decreased expression of alpha-calcium calmodulin kinase II, which is consistent with electrophysiological findings demonstrating that NMDA and AMPA currents are decreased at 7 days post-injury [19]. In most studies, the current levels used to tetanize the hippocampal slices for LTP are 40-50% of those needed for maximum EPSP, and fewer fibers are stimulated in TBI animals due to the maximum elicited EPSP being lower in TBI hippocampal slices [20–22]. Therefore, shifting the I/O curve in our data to the right may relate to impaired glutamatergic transmission and LTP in TBI animals may be difficult to induce and maintain. Fluid percussion injury at 2 Psi, which is a very low level of injury, also elicited profound but reversible suppression of PPR and LTP. This suggests that even low levels of concussive injury may have significant effects on hippocampal mechanisms which have been related to learning and memory.

### LTP CA1 Schaffer collateral mechanisms

The major components of CA1 LTP formation include glutamatergic transmission, NMDA glutamate receptors, and Ca2+ channels, which are normally blocked by magnesium (Mg2+) but which open when glutamate activates the NMDA receptors, with one important qualifier [23]. Traditionally, LTP from CA1 Schaffer collateral stimulation induced by NMDA receptors was thought to be activated by the membrane of the post synaptic neuron being partially depolarized coincident with presynaptic input, with neurotransmitter and voltage dependent Ca2+ channels then being activated [24]. Recent studies, however, have indicated that much more complex mechanisms are involved in LTP, including changes in gene expression and protein synthesis; of particular importance is the induction of CREB, PKA, and MAPK signaling pathways and synthesis of postsynaptic AMPA receptors [25–28]. In addition, there are both pre and postsynaptic structural changes at the level of dendritic spines [29, 30].

### The factors that affect short-term plasticity formation (PPF)

Paired-pulse stimulation in CA1 Schaffer collateral synapses was used to determine presynaptic neurotransmission, and our data (Figures 1 and 3) indicate that short-term synaptic plasticity was profoundly suppressed by TBI followed by KA-induced seizures. Short-term synaptic plasticity is primarily a presynaptic function that is influenced by several factors [31]. First, the role of presynaptic calcium signaling in many use-dependent forms of plasticity are important [32, 33]. Action potentials arrive at the presynaptic terminal and induce calcium influx and calcium binding to multiple low-affinity sites on synaptotagmin that trigger vesicle fusion. Therefore, short-term plasticity appears to depend primarily on the fluctuations and homeostasis in calcium levels at the synaptic site.

### Synaptic plasticity, TBI, and seizures

There are many possible causes of synaptic plasticity deficits exacerbated by seizures after TBI.

1. **Altered calcium homeostasis:** Animal studies have revealed that Ca^2+^ homeostasis in TBI-impacted hippocampal neurons is altered, a change which may result from necrotic or apoptotic cell death and abnormalities in Ca^2+^ influx and efflux. In addition, long-term changes in Ca^2+^ buffering or Ca^2+^ sequestration/release mechanisms may underlie these changes in Ca^2+^ homeostasis after TBI [34, 35]. Moreover, seizures have also been found to alter hippocampal neuron calcium homeostasis [36, 37]. Relatedly, FPI has been found to induce impairment of neurotransmission, including the transmission of dopamine and acetylcholine [38, 39], as well as glutamate. These findings may explain why FPI exacerbates the synaptic plasticity impairment caused by a KA-induced seizure.
2. **Neuron loss in the hippocampus:** Hippocampal involvement in PTE has been reported to include reduced bilateral hippocampal volume in humans after TBI [40–42]. In addition, extensive cortical and subcortical neuronal loss has also been reported after TBI [43–45]. The hippocampal CA3 and hilus have been found to be damaged initially after cortical impact [46, 47]; which results, in turn, in neuronal loss resembling “hippocampal sclerosis”, a hallmark of temporal-lobe epilepsy [45, 48].
3. **Altered synapse strength:** Time-dependent changes in synaptic proteins occur well after levels of oxidants peak following brain injury, which suggests that the depletion of antioxidant systems following trauma affects synaptic functioning and plasticity [49].
4. **Suppression of excitatory transmission:** The long-term depression of excitatory neurotransmission due to alterations in synaptic GluR2-containing, calcium-impermeable AMPARs after head injury may contribute to cognitive deficits resulting from TBI [50].
5. **In addition, an early post-TBI seizure promotes epileptogenesis and also profoundly suppresses synaptic plasticity:** The early onset of post-TBI seizures affected the severity and duration of those seizures, which may contribute to epileptogenesis [13]. However, the infusion of prophylactic levetiracetam could provide significant neuroprotection, a finding which was also previously documented [13, 51]. Such infusions may protect the cellular structure of CA1 Schaffer collateral synapses and preserve not only short-term but also long-term synaptic plasticity [52, 53].

The antiepileptic effects of LEV may involve several mechanisms [51, 52]: (1) interaction with SV2A which is one isoform of synaptic vesicle protein 2 (SV2) (2) removal of the Zn++-induced suppression of GABA-mediated presynaptic inhibition. (3) the combination of inhibitory effects on depolarization-induced and Ca2+ release-associated neurotransmitter releases. (4) modulation of the presynaptic P/Q-type voltage-dependent calcium (Ca2+) channel to reduce glutamate release in the dentate gyrus of the hippocampus that regulates seizure activities. (5) via intracellular inhibition of presynaptic Ca2+ channels to inhibit neurotransmitter release. Furthermore, daily LEV treatment may have beneficial effects on the histological, molecular, and behavioral elements of neurological recovery after TBI, in part, via modulation of neuroinflammatory and excitatory pathways.

## MATERIALS AND METHODS

### Ethics statement

6-week-old male Sprague-Dawley rats (N=40) were used in the present study. The animals were housed under a 12 hr light/dark cycle and provided with food and water ad libitum. All animal procedures were conducted in accordance with NIH guidelines and were reviewed and approved by the Institutional Animal Care and Use Committee (IACUC) of the National Defense Medical Center (Taiwan Protocol Number IACUC-13-145). The number of animals used was the minimum number required, based on power calculations (α=0.05 and 1-ß=0.80). All surgery was done under aseptic conditions and postoperative analgesia was provided by administration of NSAIDs. Perioperative opiates were not used as they have been shown to be neuroprotective and might have confounded interpretation of the results.

### Experimental protocols and groups of animals (Supplementary Data 1)

Two separate experiments were performed to examine the effect of single and repeat seizures, respectively. In the first experiment, rats underwent fluid percussion injury at 2 Psi and were subsequently induced to have a single seizure at 1 hour (FPI+1hr, N=3), 3 days (FPI+3d, N=4), or 7 days after TBI (FPI+7d, N=4), and were compared with control animals not exposed to TBI (control, N=5). For the second experiment, rats underwent fluid percussion injury followed by induction of three seizures at weekly intervals starting at 1 week after TBI (FPI+ES, N=3), 2 weeks after TBI (FPI+LS, N=3), and 1 week after TBI with prophylactic levetiracetam infusion (25.2 mg/kg/day) via ALZET mini-pump for 7 days (FPI+LEV+ES, N=3); these were compared with uninjured rats (control, N=5), rats exposed to TBI but not induced to have seizures (FPI-saline, N=3), and rats not exposed to TBI but induced to have three seizures at one week intervals (Sham-ES, N=3).

### Seizure induction

Kainic Acid (Sigma-Aldrich, Co., St Louis, MO, US, 7 mg/kg) was injected via the tail to induce seizures in each of the seizure groups. All the animals underwent electrode implantation into the M1 cortex to further identify the onset of seizures and the epileptic spikes. To control for postictal apnea, all the animals received manual artificial respiration via a tube placed over the nose until spontaneous respiration was seen. The mortality rate in each group with three times of KA-induced seizure protocol were: FPI-ES: 3/6 (50%); FPI-LS: 0/3; Sham-ES: 0/3; FPI-LEV-ES: 0/3; and total mortality rate was 3/15 (20%).

### Fluid percussion traumatic brain injury

The fluid percussion device (model HPD-1700, Dragonfly R&D, USA) used to produce TBIs in the rats has previously been described by Matsushita et al.[54] The specific methods used to produce midline FPIs are detailed in our previous papers [55, 56] but are briefly reiterated here to facilitate reproduction of our results. Animals were anesthetized using Zoletil 50mg/kg IP; a 4.8 mm diameter burr hole was then drilled at the midline between the coronal and lambdoid sutures, and a Leur-Loc hub was affixed to the perimeter of the burr hole using cyanoacrylate. Dental acrylic and two small nickel-plated screws were then used to anchor the hub to the skull. The rats were anesthetized, the surgical site exposed, and the animal was connected to the injury device. Injury was induced by striking the piston with a weighted metal pendulum released from a pre-determined height or angle. The resulting rapid injection of a small volume of saline into the closed cranial cavity produces a pulse of increased intracranial pressure that is associated with a deformation of the brain. The force of the injury administered was 2 Psi of pressure and resulted in the suppression of the righting reflex for a period of between 6 and 9 min. Sham animals were connected to the injury device but no injury was delivered, and suppression of the righting reflex lasted less than 60 sec. Pressure pulses were measured extra-cranially with a pressure transducer, recorded on a digital real-time oscilloscope (TDS210, Sony Tektronix Corp., Japan), and analyzed by Wave Star software (Sony Tektronix Corp., Japan). The fluid percussion device delivered transient pressure fluid pulses with constant wave form and duration (17–21 ms) to cause brain injury [57].

Prophylactic levetiracetam were administered by infusion pump implanted after TBI for one week (Infusion of LEV (25.2mg/kg/day) via ALZET mini-pumps for 7 days after TBI. The LEV+FPI+ES group received levetiracetam infusion pump implantation right after FPI for one week and the KA induced seizures were performed after we removed the pump. The control group for these animals were TBI + KA without LEV. This is also shown in Supplementary Data 1. Many of the other details on the control groups, LEV administration, and timing were detailed in our previous paper [13]. The animals used here on hippocampal plasticity, which is new, were a subset of those animals.

### Hippocampal brain slice preparation

Hippocampal slices from the rat brains were prepared according to the procedure described in previous reports [58, 59]. Each animal was decapitated and its brain quickly removed and rapidly immersed in cold (4°C), oxygenated, high-sucrose, low-Ca2+-containing artificial cerebrospinal fluid (aCSF) with the following composition: 87 mM NaCl, 2.5 mM KCl, 7 mM MgCl2, 0.5 mM CaCl2, 1.25 mM NaH2PO4, 25 mM glucose, 75 mM sucrose, and 25 mM NaHCO3. Using a vibrating tissue slicer (VT1000S; Leica Instruments), the brain was sliced into 300–350-μm thick coronal slices. Incubation of the hemisectioned brain slices containing the hippocampus in normal aCSF consisting of 126 mM NaCl, 3.0 mM KCl, 1.5 mM MgCl2, 2.4 mM CaCl2, 1.2 mM NaH2PO4, 11.0 mM glucose, and 25 mM NaHCO3 saturated with 95% O2 and 5% CO2 at room temperature was then carried out for ≥90 min before electrophysiological recordings of the slices were made. After being transferred to a recording chamber, individual brain slices were continuously superfused with normal aCSF (2 mL/min) and maintained at a temperature of 30°–32°C using a temperature-controlled solution heater (TC-324B; Warner Instruments). A syringe pump (Razel) was then utilized to deliver drugs to the slices via superfusion.

### *In vitro* hippocampal electrophysiology

Following preparation procedures described in previous studies [58, 60], field excitatory postsynaptic potentials (fEPSP) of each individual brain slice were recorded, with all of the recordings being conducted in the CA1 region of the hippocampus. Specifically, extracellular fEPSPs were obtained using 3 M NaCl-filled electrodes and an AC amplifier (A-M Systems Model 1800), with the signals being high- (10 Hz) and low-pass (10 kHz) filtered. Using an A/D board (National Instruments PCI 6024E, or Digidata 1320A; Axon Instruments), the fEPSP data were transmitted at 4 kHz to a personal computer running Windows-based software (WCP, courtesy of John Dempster, University of Strathclyde, Glasgow, UK; http://www.strath.ac.uk/sipbs/; or pCLAMP 9.0, Axon Instruments). Using single, 0.1-msec pulses, delivered at a frequency of 0.033 Hz through a bipolar electrode constructed with formvar-insulated nichrome wire, responses were elicited from each brain slice via electrical stimulation of the stratum radiatum. The intensity of these stimuli was adjusted as necessary to yield fEPSPs with peak amplitudes of 0.5–1 mV (30%–40% of the maximal response to avoid ceiling effects). Prior to electrical stimulation, at least 10 min of stable baseline recordings were obtained. Using the data acquisition software, the peak amplitude and slope of the initial (1–2 msec) rising phase of each fEPSP were calculated off-line, with any changes to the synaptic response being normalized to the aforementioned baseline period. High-frequency stimulation (HFS), consisting of three 1-sec trains of 100 Hz delivered at 10-sec intervals at twice the stimulation intensity utilized to evoke LTP was then administered. Following these HFS trains, the stimulation intensity was then returned to the level previously utilized to produce baseline fEPSPs.

### Statistical analysis (Supplementary Data 2)

Data in the text and figures are expressed as means + SEM. Statistical analyses of data for the dopamine release input/output curves, fEPSP slope curves, and paired-pulse ratio were performed using a two-way analysis of variance (ANOVA) followed by a Bonferroni post hoc test for multiple comparisons. All statistical tests were two-tailed and were performed using GraphPad Prism 5.02 (GraphPad Scientific, San Diego, CA, USA). A p-value < 0.05 was considered significant for all analyses. ^*^p<.05, ^**^p<.01, ^***^p<.001 in all figures.

## CONCLUSIONS

In this study, we investigated short-term and long-term synaptic plasticity suppression after TBI and KA-induced seizures. Our data indicate that general synaptic (presynaptic and postsynaptic) suppression occurs in TBI animals experiencing a subsequent seizure. Such synaptic suppression affects neurotransmission in terms of both PPF or LTP, with the presynaptic and postsynaptic transmission suppression after a seizure alone being transient, whereas such transmission suppression persists for a longer time when a seizure is combined with a TBI. Moreover, prophylactic levetiracetam infusion partially reverses such long term suppression of synaptic plasticity.

## SUPPLEMENTARY MATERIALS FIGURES AND TABLES



## Abbreviations

2 Psi: 2 Pounds per square inch
aCSF: artificial cerebrospinal fluid
AMPARs: AMPA receptors
ANOVA: Analysis of variance
CA1: cornu ammonis 1
Ca: Calcium
CA3: cornu ammonis 3
EEG: Electroencephalography
fEPSP: field excitatory post synaptic potentials
FPI: fluid percussion injury
HFS: High-frequency stimulation
I/O curves: input-output curves
IACUC: Institutional Animal Care and Use Committee
IPI: interpulse intervals
KA: kainic acid
LEV: levetiracetam
LTP: long-term potentiation
M1 cortex: primary motor cortex
Mg: magnesium
NSAIDs: nonsteroidal anti-inflammatory drugs
PPF: paired pulse facilitation
PPR: paired-pulse ratio
PTE: posttraumatic epilepsy
TBI: traumatic brain injury.
