# Supplementary material for: Profound deficits in hippocampal synaptic plasticity after traumatic brain injury and seizure is ameliorated by prophylactic levetiracetam
Source: Oncotarget. 2018 Jan 4;9(14):11515–27. doi: 10.18632/oncotarget.23923 (PMC5837755; doi:10.18632/oncotarget.23923)
Supplement: Supplementary file 1 [file oncotarget-09-11515-s001.pdf]

Profound deficits in hippocampal synaptic plasticity after traumatic brain injury and seizure is ameliorated by prophylactic levetiracetam

SUPPLEMENTARY MATERIALS

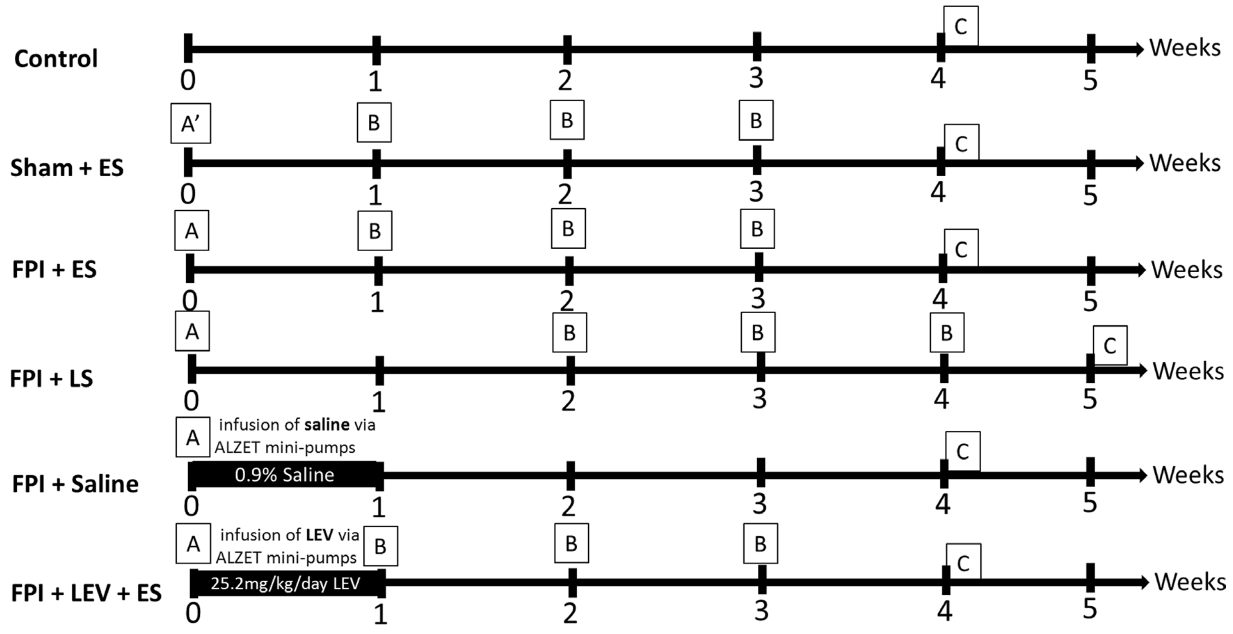

Supplementary Figure 1:
